# Double Primary Cancer of the Prostate and Urothelial Cancer: A Single Institution Experience

**DOI:** 10.3390/jpm14050510

**Published:** 2024-05-11

**Authors:** Senji Hoshi, Vladimir Bilim, Kiyotsugu Hoshi, Yoshihiro Ogawa, Tomoyuki Kato, Kota Urano, Tomoya Yamada, Rie Sakagami, Takashi Kudo, Kenji Numahata, Isoji Sasagawa

**Affiliations:** 1 Department of Urology, Yamagata Tokushukai Hospital, Yamagata 990-0834, Japan; senjihoshi47@yahoo.co.jp (S.H.); kiyotsugu49@yahoo.co.jp (K.H.); isoji.sasagawa@tokushukai.jp (I.S.); 2 Department of Urology, Kameda Daiichi Hospital, Niigata 950-0165, Japan; 3 Sendai Radiation Oncology & Imaging Clinic, Sendai City 981-3121, Japan; ogaway@sendai-roic.or.jp; 4 Department of Urology, Yamagata City Hospital Saiseikan, Yamagata 990-8533, Japan; tomoyuki.kato@saiseikan.jp; 5 Department of Urology, Yamagata Prefectural Central Hospital, Yamagata 990-2292, Japan; kotakoura4@gmail.com (K.U.); t.yam.lynxciliegio@gmail.com (T.Y.); rier060209@gmail.com (R.S.); takashi0218kudo@yahoo.co.jp (T.K.); knumapyon@gmail.com (K.N.)

**Keywords:** prostate cancer, bladder cancer, urothelial cancer, ureteral cancer

## Abstract

Prostate cancer (PCa) ranks as the second most common cancer in Japanese males, while bladder cancer (BC) holds the tenth spot. Among double urological cancers, the incidence of synchronous or metachronous BC and PCa is the highest. Reports on upper urinary tract (UUT) urothelial cancer (UC) in PCa patients are limited. Here, we present three cases of metachronous PCa and BC, with subsequent diagnosis of ureteral and renal pelvic cancer during the course of the disease. In the follow-up of patients with urological cancers, it is important to be aware not only of the progression of the initial cancer but also the potential development of a second cancer.

## 1. Introduction

With the advent of anticancer treatment modalities, increasing numbers of cancer patients are achieving long-term survival. Cancer is associated with increased age, and cancer survivors have a higher risk of the development of secondary cancer. The development of secondary cancer can be associated with primary cancer treatment such as radiotherapy or chemotherapy. A common genetic background can determine susceptibility to both cancers. Moreover, exposure to certain environmental carcinogens may play a role in the development of primary and secondary cancers. Research has shown that different primary cancers can correlate with specific secondary cancers. For example, patients with bladder cancer (BC) have an increased risk of prostate cancer (PCa). Thus, such patients may benefit from increased surveillance [1]. Patients with PCa have about a 10% risk of developing secondary cancer, making it the third most common cancer in this aspect, following the patients with lung and colorectal cancer [2].

Prostate-specific antigen (PSA) screening results in the increased detection of localized PCa. A significant portion of PCa cases are diagnosed at stages 1 or 2. In the US, it has been reported that about 60% of PCa cases were diagnosed as localized cancer, while approximately 30% were diagnosed as locally invasive PCa or PCa with metastasis to regional LNs. Only about 10% of newly diagnosed PCa cases have distant metastases at the time of diagnosis. Treatment options for PCa include surgery, various radiation therapy modalities, including high- and low-dose brachytherapy, IMRT (intensity-modulated radiation therapy), proton therapy, heavy ion therapy, hormone therapy, and watchful waiting, among others. The choice of treatment depends on factors, such as the stage of cancer, overall health, and patient preferences. CyberKnife [3] is a robotic radiotherapy device that utilizes a stereotactic body radiation therapy approach. It operates with extreme accuracy, reducing the exposure of surrounding normal tissues to ionizing radiation. This has the potential to prevent the development of secondary cancers.

Androgen deprivation therapy (ADT) is a standard of care for metastatic prostate cancer (PCa); however, patients inevitably progress to castration-resistant prostate cancer (CRPC) when the treatment options are limited. Median overall survival with abiraterone was reported to be 20.9 months, and it was improved to 22.7 months with olaparib and abiraterone [4].

Urothelial cancer (UC) is the most common cancer of the bladder, accounting for 90–95% of cases. It has been reported that about 90% or more of UCs originate in the bladder. The remaining 5–10% originate in the upper urinary tract (UUT). Approximately 5–7% of UCs occur in the renal pelvis, and 1–2% occur in the ureter. The standard of care for localized UC is surgical treatment (transurethral resection of the bladder tumor or nephroureterectomy for UUT cancer).

Incidence rates for both cancers increase with age, and the risk of developing synchronous or metachronous PCa or BC in patients with another diagnosed type of cancer is 18 times higher than that of the general population. Double PCa and BC can be a result of the increased incidence of each cancer in elderly males. However, there is evidence to suggest a molecular association between these two cancers. External beam radiation therapy and brachytherapy for PCa increase the risk of secondary bladder cancer [5,6,7].

Although many studies have shown an association between PCa and BC, there are only a few reports about PCa and UUT UC.

## 2. Case Presentation

**Figure 1:**
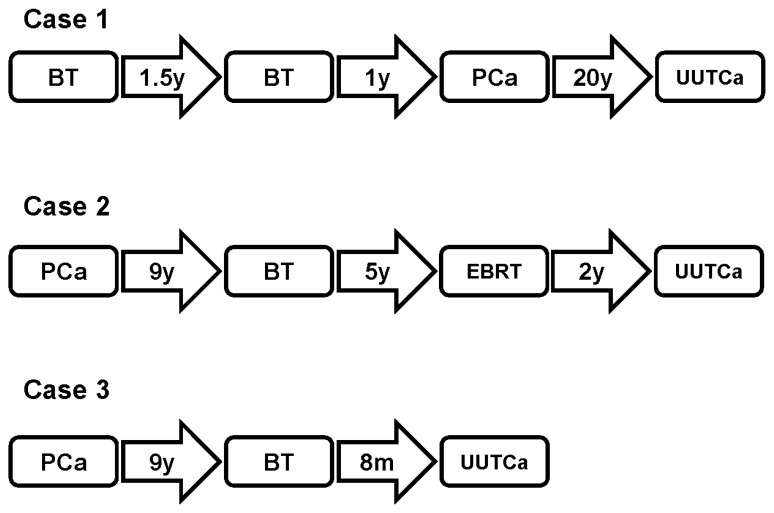
The treatment timeline.

The three patients were diagnosed with metachronously developed PCa and UUT UC. PCa was initially treated with a radical retropubic prostatectomy (RRP). The patients were examined using a gene mutation panel (GenMineTOP Cancer Genome Profiling System, Konica Minolta REALM, Inc. Tokyo, Japan) and no mutations were found in cancer-associated genes. Diffusion-weighted whole-body imaging with background body signal suppression (DWIBS) was used as an imaging technique for the follow-up of patients. Patients’ clinicopathological data are summarized in Table 1, and the treatment timeline is presented in Figure 1.

### 2.1. Case 1

A 58-year-old patient was diagnosed with bladder cancer and underwent a transurethral resection (TURBT) in August 2000. One and a half years later, a second TURBT was performed due to bladder recurrence. One year following this, he presented with an elevated PSA of 6.8 ng/mL, and a Gleason Score (GS) of 3+4 PCa was identified via a prostate biopsy. Subsequently, he underwent a radical retropubic prostatectomy (RRP) in January 2004, with the pathological diagnosis revealing pT2bN0 and a GS of 4+4.

Six years post-RRP, his PSA rose to 0.94 ng/mL, prompting treatment with a luteinizing hormone-releasing hormone (LHRH) agonist in conjunction with bicalutamide, which was later switched to flutamide. Despite this, his PSA continued to rise sequentially to 1.36, 1.45, and 2.34 ng/mL, leading to a diagnosis of castration-resistant prostate cancer (CRPC).

The patient underwent sequential treatments with abiraterone, docetaxel, cabazitaxel, darolutamide, and estramustine. Subsequently, bone metastases were detected, and he received radium-223 therapy three times, with the final dose administered in June 2023.

**Figure 2:**
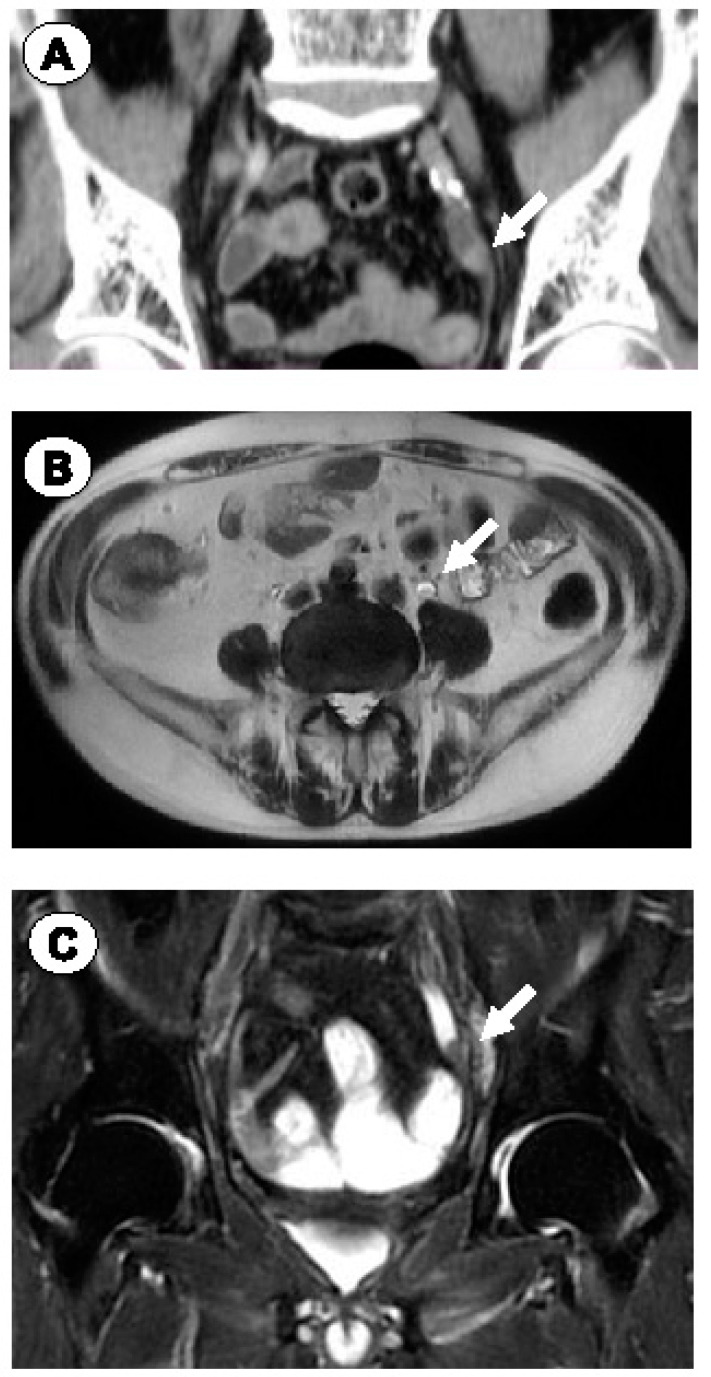
Case 1. Coronal CT (**A**), and axial (**B**) and coronal (**C**) MRI images demonstrating a left ureteral tumor. Arrows indicate the tumor.

At the age of 81 years, in July 2023, a left ureteral tumor was discovered (Figure 2), and the patient underwent a left ureteral resection with neoimplantation into the bladder. While this procedure is not standard for ureteral urothelial carcinoma, it was considered in this elderly patient who had undergone extensive prior treatments. He subsequently developed lung hilar lymph node metastases, the origin of which could either be PCa or urothelial cancer, which were treated with CyberKnife.

### 2.2. Case 2

A 75-year-old patient had an elevated PSA of 9.3 ng/mL and a Gleason Score (GS) of 3+4 PCa identified via a prostate biopsy. He underwent a radical retropubic prostatectomy (RRP) in November 2006, and the pathological diagnosis was adenocarcinoma with a GS of 3+4, staged as pT3aN0. A maximum androgen blockade (MAB) with leuprolide and casodex was started for locally invasive PCa, and intermittent hormone therapy was performed at the patient’s request. There was no biochemical recurrence, with the PSA ranging from 0.71 to 1.685 ng/mL.

In October 2015, 9 years after the RRP, the patient complained of asymptomatic macrohematuria, and multiple small pedunculated papillary tumors were detected via a cystoscopy, which were successfully resected by transurethral resection (TUR). The pathological diagnosis was low-grade non-invasive urothelial cancer (UC).

Five years later, in October 2020, his PSA increased to 5.021 ng/mL, and a follow-up cystoscopy revealed a tumor in the bladder neck, which was treated by TUR. The pathological diagnosis was PCa with a GS of 4+4. The patient underwent external beam irradiation of the prostate bed (70 Fr in 35 fractions).

**Figure 3:**
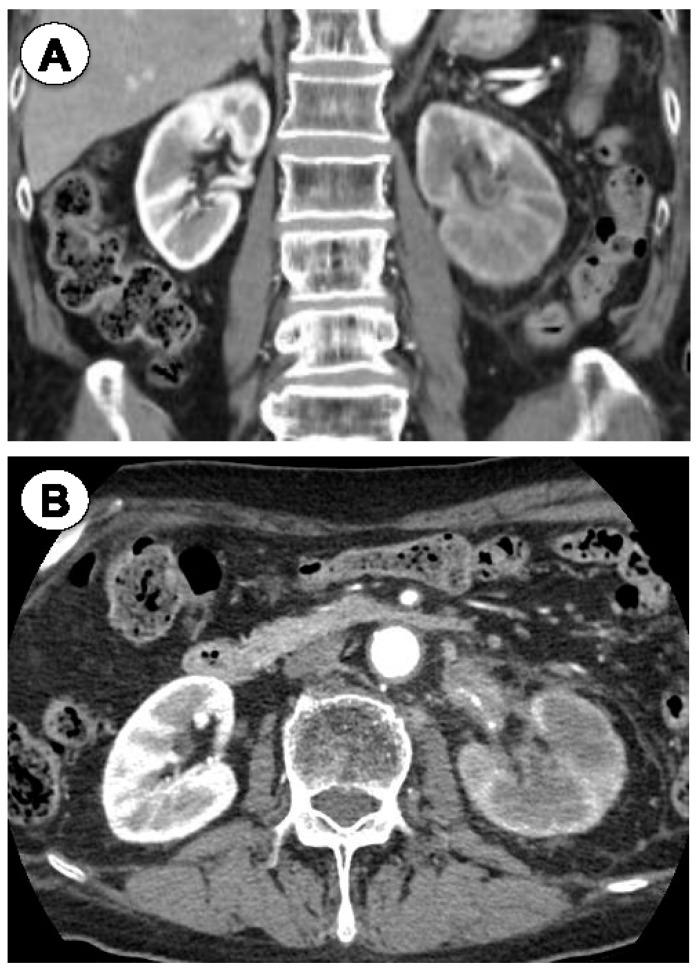
Case 2. Coronal (**A**) and axial (**B**) CT images demonstrating invasive left renal pelvic cancer.

Two years later, in November 2022, his urinary cytology was class III, and invasive left renal pelvic cancer was detected by CT (Figure 3), which was surgically resected in October 2022. The diagnosis of invasive UC was confirmed by pathology, and nivolumab treatment was started for invasive UC with a retroperitoneal lymph node metastasis.

The patient passed away due to disease progression seven months later at the age of 92 years old.

### 2.3. Case 3

A 61-year-old patient had an elevated PSA of 5.8 ng/mL and a Gleason Score (GS) of 4+3 PCa identified via a prostate biopsy. He underwent a radical retropubic prostatectomy (RRP) in November 2014, and the pathological diagnosis was adenocarcinoma with a GS of 4+4, staged as pT2bN0. There was no recurrence of PCa during the follow-up.

Nine years later, a cystoscopy was performed due to asymptomatic macrohematuria, which revealed a bladder tumor. The tumor was treated by TURBT, and the pathological diagnosis was low-grade UC.

**Figure 4:**
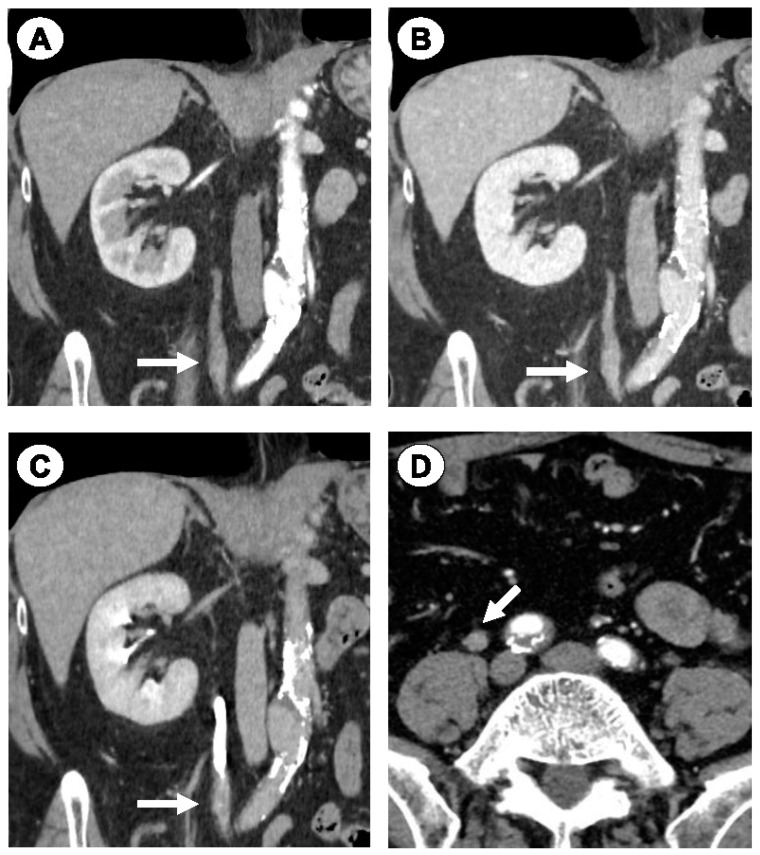
Case 3. Dynamic CT images demonstrating a right ureteral tumor. Coronal view of renal cortex enhancement (**A**), and parenchymal (**B**) and excretion phases (**C**). Axial view (**D**). Arrows indicate the tumor.

Eight months later, right ureteral carcinoma was suspected based on follow-up CT findings (Figure 4). Urinary cytology indicated class IIIa. The patient underwent a right nephroureterectomy, and the pathological diagnosis confirmed urothelial carcinoma (UC). The patient is currently disease-free three months after the operation.

## 3. Discussion

Urologic cancer burden has increased globally with the general population aging. This can cause an increased incidence of double PCa and UC in the same person. However, it has been reported that the coincidence rate between BC and PCa is significantly higher than the expected diagnosis frequency based on disease prevalence in the general population [6,8,9]. PCa survivors have a greater risk of bladder cancer [7]. Detecting one genitourinary tumor can lead to a more extensive clinical assessment and regular clinical follow-ups, causing diagnostic bias.

Several factors can explain the positive association between the two cancers, such as environmental or genetic risk factors, lifestyle (e.g., tobacco, alcohol, and diet), the environment (e.g., contaminants and occupation), and host factors (e.g., genetics, immune function, and hormonal interactions), which can affect the carcinogenesis and development of double cancers, modulating the risk of double cancer in the same person. Micronutrients play a vital role in the development of urological tumors [10]. There is a possibility that micronutrients can independently cause PCa and UC in the same person. Decreased vegetable consumption can be associated with bladder cancer and PCa [11]. Occupational exposure to pesticides is associated with BC and PCa [12]. Diabetes has been attributed to an increased risk of several cancers, including PCa and bladder cancer. The three patients in this series did not have a history of diabetes.

However, the prevalence of incidental PCa after a radical cystectomy for bladder cancer ranged from 20 to 60% [13,14,15], with less aggressive PCa diagnosed in Asia than in Europe or the United States. In a large radical cystectomy series, a 20% rate of incidental PCa was reported and the majority of PCa cases had clinically insignificant tumors [16]. These findings align with previously published results of 26.4% of incidental prostatic carcinoma found at autopsy [17].

There is a possibility of shared genetic risk factors related to mutations in a proto-oncogene. Lynch syndrome with mutations in the genes MLH1, MSH2, MSH6, and PMS2 can be the cause of both UC and PCa [18]. Male BRCA mutation carriers have an increased risk of both PCa and UC [19]. However, in sporadic double PCa and BC, different patterns of mutation combinations within the two tumors were observed, suggesting that different genetic mutations worked for tumorigenesis in each case [20]. Deregulation of long non-coding RNAs [21] or circular RNAs [22] has also been attributed to the development of multiple cancers. In these patients, no mutations were found using a gene mutation panel, and no family history of UC or PCa was noted.

Radiation therapy for PCa is associated with an increased risk of urinary bladder cancer [5,6,7]. However, the latency period between radiation exposure and radiation-induced malignancies is estimated to be more than 5 years. Moreover, patients treated with RT are often older, which, by nature, possibly increases the risk of bladder cancer. In these three patients, EBRT was not performed before the diagnosis of BT. It has been shown that radiation therapy with the CyberKnife System produces excellent control with low toxicity in patients with low–intermediate-risk prostate cancer [3]. In this series, CyberKnife was not used for the local treatment of prostate cancer, but it was utilized to treat lymph node metastases in patient 1.

There is one interesting study that demonstrated the protective role of androgen deprivation therapy for intravesical recurrence of nonmuscle invasive bladder cancer [23]. Androgen receptors have been shown to be expressed in bladder epithelium as well as in bladder cancer tissues. Androgen receptor signaling has been reported to promote DNA breaks and chromosomal rearrangements, and it suppresses detoxification and the elimination of carcinogens. The connection between androgen receptor signaling and urothelial cancer can explain the male predominance of this type of cancer. Thus, ADT in PCa patients can decrease the risk of secondary bladder cancer.

In this small series, all three patients presented with bladder cancer followed by a diagnosis of ureteral cancer, with the longest time between the two diagnoses being 22 years. In patient 1, BC preceded the diagnosis of PCa, and in the remaining two patients, PCa was diagnosed first.

Bladder UC and UUT UC share many similarities, including histology and risk factors. Molecular similarity between the two cancers has been reported [24]. However, other studies provide evidence for molecular differences between UUT and bladder UC [25]. Patients with UUT UC are at increased risk of recurrence in the bladder. This can be explained by anatomical connectivity and the shedding of tumor cells from the UUT into the bladder. However, all three patients were first diagnosed with BC and then with ureteral cancer. Several clinicopathological factors have been established as risk factors for UC recurrence. These include the number of tumors, tumor size and grade, presence of concomitant CIS, and smoking history. However, BC in all three patients was low-stage and low grade one, and no CIS was found. The increased risk of UUT UC in patients with bladder UC is multifactorial, involving shared genetic pathways, common risk factors, anatomical connectivity, and the effects of diagnostic surveillance. The BT and UUT relationship can be explained by a field effect, which is thought to be a field change that affects the entire urothelium.

## 4. Conclusions

A higher level of vigilance is required for PCa in patients with BC, and vice versa. This is both true for the primary evaluation of a patient and follow-up after curative treatment. Patients with BC should be screened with PSA, and hematuria after PCa treatment should raise concerns about possible UC. Regular diagnostic imaging using CT or MRI should be performed in PCa patients to both detect metastatic disease and evaluate the UUT for UC.

## Figures and Tables

**Table 1:** Patients’ clinicopathological data.

	Age at Presentation	iPSA	RRP GS	Time until Secondary Cancer	Time until UUT Cancer	Outcome
1	58 y	6.8 ng/mL	4+4	2 y	22y	CRPC
2	75 y	9.3 ng/mL	3+4	9 y	16y	Dead with UUT cancer
3	61 y	5.8 ng/mL	4+4	9 y	10y	NED

## Data Availability

The data that support the findings of this study are available on request from the corresponding author, (V.B.).

## Abbreviations

BC	bladder cancer
CRPC	castration-resistant prostate cancer
GS	Gleason score
iPSA	initial PSA
MAB	maximal androgen blockade
PCa	prostate cancer
PSA	prostate-specific antigen
TUR	transurethral resection
UC	urothelial cancer
UUT	upper urinary tract
